# The role of *Wnt *signaling in neuronal dysfunction in Alzheimer's Disease

**DOI:** 10.1186/1750-1326-3-9

**Published:** 2008-07-24

**Authors:** Nibaldo C Inestrosa, Enrique M Toledo

**Affiliations:** 1 Centro de Envejecimiento y Regeneración (CARE), Centro de Regulación Celular y Patología "Joaquín V. Luco" (CRCP), MIFAB, Facultad de Ciencias Biológicas, Pontificia Universidad Católica de Chile, Alameda 340, Santiago, Chile; 2 CARE & CRCP Biomedical Center, Faculty of Biological Sciences, P. Catholic University of Chile, P.O. Box 114-D, Santiago, Chile

## Abstract

Recent evidence supports a neuroprotective role for *Wnt *signaling in neurodegenerative disorders such as Alzheimer's Disease (AD). In fact, a relationship between amyloid-β-peptide (Aβ)-induced neurotoxicity and a decrease in the cytoplasmic levels of β-catenin has been observed. Apparently Aβ binds to the extracellular cysteine-rich domain of the Frizzled receptor (Fz) inhibiting *Wnt*/β-catenin signaling. Cross-talk with other signaling cascades that regulate *Wnt*/β-catenin signaling, including the activation of M_1 _muscarinic receptor and PKC, the use of Ibuprofen-ChE bi-functional compounds, PPAR α, γ agonists, nicotine and some antioxidants, results in neuroprotection against Aβ. These studies indicate that a sustained loss of *Wnt *signaling function may be involved in the Aβ-dependent neurodegeneration observed in Alzheimer's brain. In conclusion the activation of the *Wnt *signaling pathway could be proposed as a therapeutic target for the treatment of AD.

## Introduction

Alzheimer's disease (AD) is a neurodegenerative disorder associated with aging and characterized by fibrillar deposits of Aβ in subcortical brain regions. Typical features of AD are extracellular neuritic amyloid plaques (senile plaques) and intracellular neurofibrillary tangles. The main proteinaceous component of the amyloid deposited in AD is the Aβ peptide, a 40-to 42-residue peptide that has been isolated from senile plaque cores. Studies in AD mouse models and AD patients support the hypothesis that Aβ causes "synaptic failure" before plaques develop and neuronal cell death occurs; such effects are produced by Aβ oligomers, which are soluble and toxic molecular forms of Aβ [1].

The importance of *Wnt *(wingless-type murine-mammary-tumour virus integration site) signaling in many adult and developmental processes, such as gastrulation, axis formation, cell polarity, organ development and maintenance of stem cell pluripotency, is widely acknowledged [2,3]. In embryos, signaling by *Wnt *factors controls the organization of the body plan during the early stages of development as well as organogenesis at later developmental stages. Postnatally, *Wnt *signaling is involved in normal biological events such as tissue maturation and homeostasis and in several neoplastic pathologies. In the mammalian central nervous system (CNS), *Wnt *signal transduction is involved in neural induction and patterning in early embryogenesis; previous studies have also linked *Wnt *signaling to neurodegenerative disorders such as AD [4-6]. In fact, strong evidence suggests that a loss of *Wnt *function is implicated in the pathophysiology of neuronal degeneration of AD. *Wnt *signaling is complex; 19 mammalian *Wnt *genes have been cloned, and more than ten membrane receptors and a plethora of cofactors and regulators are known. Different mechanisms of *Wnt *signaling have also been identified. The best understood of these is the "canonical" pathway, in which β-catenin transduces the *Wnt *signal to the nucleus [7]. In this case, the signaling cascade by *Wnts *involves an interaction with a receptor complex comprising members of the Frizzled (Fz) class of 7-transmembrane receptors and a member of the low density lipoprotein receptor 5/6 (LRP 5/6) family of single-pass membrane proteins. *Wnt *interaction with its receptor results in an increase in the stability of β-catenin, whose accumulation results in translocation to the nucleus where it can interact with members of the TCF/LEF class of transcription factors and therefore modulate gene expression. The stability of β-catenin is controlled by *Wnt *through the modulation of a large cytoplasmic protein complex comprised of the protein Axin (axis inhibition protein), APC (adenomatosis polyposis coli), CK1α (casein kinase 1 alpha), GSK-3β(glycogen synthase kinase 3 beta) and GβP/frat [8]. GSK-3β directly controls the level of β-catenin phosphorylation, which leads to its consequent degradation by the proteasome pathway [9]. *Wnt *signaling is regulated by a wide range of proteins, which act either intracellularly by affecting signal transduction, or extracellularly by interfering with the interaction between *Wnt *ligands and their membrane co-receptors [10]. Different families of extracellular antagonists of the canonical *Wnt *pathway have been described, such as Wise, the secreted frizzled-related protein (sFRP), the *Wnt *inhibitory factor 1 (Wif1), Cerberus, and the Dickkopf (Dkk) family of secreted proteins. Of the four known Dkk family members, Dkk-1 is uniquely described as a negative modulator of the canonical *Wnt *signaling, whereas, Dkk-2 for example may activate or inhibit the pathway depending on the cellular context. Dkk-1 is expressed at very low levels in the adult brain [11], and binds to LRP 5/6 and the transmembrane protein Kremen-2, promoting the endocytosis and subsequent degradation of LRP 5/6, which is no longer available as a co-receptor for *Wnt *[12].

Little is known about the role of the heparan sulfate proteoglycans (HSPGs) in vertebrate *Wnt *signaling [13]. A comparable signaling system, however, may help to elucidate its involvement. Genetic evidence demonstrates that two *Drosophila *genes involved in *Wg *signaling, *dally *(division abnormally delayed) and *dlp *(dally-like), reveal a predicted protein sequence that resembles the protein cores of glypican (HSPG) [14-16]. Flies homozygous for hypomorphic *dally *alleles exhibit some wing-margin defects, a phenotype similar to partial loss of *Wg *activity [14]. *Dally*'s sensitivity to heparin lyase II and not to chondroitinase ABC treatments indicates that it contains heparan sulfate chains [16]. With this understanding, for studying the involvement of HSPG in Neuro2a cells and hippocampal neuron signaling, we used heparin as a glycosaminoglycan (GAG) model to investigate the modulation of β-catenin. We found that heparin modulates the levels of cytoplasmic β-catenin in a concentration-dependent manner in Neuro2a cells. Mainly HS residues are involved, since other GAGs, such as chondrotin (CS) or dermatan sulfate (DS), had little effect. The effect of heparin involves a decrease in the activity of GSK-3β and phosphorylation of its Ser 9 residue complemented with the increase of β-catenin. These results are consistent with the idea that increases in β-catenin levels are the result of an inhibition of GSK-3β activity, particularly through phosphorylation of the Ser 9 residue. In addition, heparin affects β-catenin and GSK-3β activity in rat hippocampal neurons, and *Wnt*-3a modulates the effect of heparin on β-catenin levels [17]. More importantly, the presence of heparin enhances the protective effect of *Wnt*-3a against β-amyloid neurotoxicity (Table 1).

**Table 1:** Heparin Modulation of the *Wnt-3a *ligand Effect on the Survival of Hippocampal Neurons Exposed to the Aβ peptide

Treatment	Cell Survival (%)
*Control*	100.0 ± 6.1
*A*β	51.4 ± 2.9
*A*β *+ Wnt-3A*	75.9 ± 4.3
*A*β *+ Wnt-3A + heparin 0.1 μg/ml*	88.3 ± 6.0 *
*A*β *+ Wnt-3A + heparin 1.0 μg/ml*	103.1 ± 8.5 *

Values represent means ± s.d. of three experiments carried out in triplicate. Hippocampal neurons were pretreated with *Wnt-3a *ligand with or without heparin for 1 h previous to the addition of 5 mM Aβ_1–40_. Neurons were then incubated for 24 h and MTT reduction was determined. * Indicates P < 0.05 compared with respect to Aβ + *Wnt-3a *in a "T" test analyzed by the Sigma plot 2.0 program.

Historically, *Wnt *proteins were classified as either canonical, such as *Wnt-1 *and *Wnt-3a*, or non-canonical, including *Wnt-4*, *Wnt-5 *and *Wnt-11 *[7,18,19]. The characterization of Fz, LRPs and other receptor function has challenged this classification of individual *Wnt *proteins. Evidence suggests that *Wnt-5a*, for example, may activate the canonical pathway or inhibit it, depending on the receptor involved [20]. Accordingly, the terms "canonical" and "non-canonical" are used to indicate molecular mechanisms, not specific *Wnt *proteins. Two non-canonical *Wnt *pathways have been described to play a role in development: (i) the planar cell polarity (PCP) pathway, in which Fz acts through Jun N-terminal kinase (JNK) to regulate the cytoskeleton, and (ii) the *Wnt-*Ca^2+ ^signaling pathway, in which Fz activation leads to increased intracellular Ca^2+ ^and nuclear import of the transcription factor NFAT [21]. These show that not only are alternative *Wnt *pathways utilized to specify pattern formation during development, but also different mechanisms. Although the final output of the canonical and the non-canonical *Wnt*-Ca^2+ ^pathways are the regulation of gene expression, the PCP pathway controls planar cell polarity by modulating the cytoskeleton [22,23].

**Figure 1:**
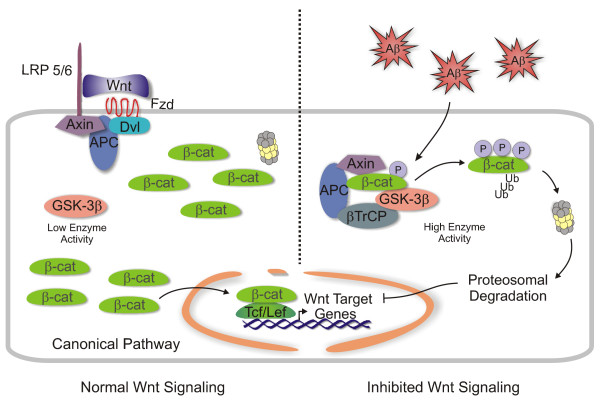
**The *Wnt *signaling pathway and its inhibition by Aβ aggregates**. First when the *Wnt *ligand is available, the Fz receptor together with LRP5/6 translates its signal through Dvl, which in turn inactivates GSK-3β in the cytoplasmic destruction complex. This allows β-catenin to accumulate in the cytoplasm, and subsequently to move to the nucleus, where it binds to TCF/LEF transcription factors activating *Wnt *target gene transcription (*Left Panel*). On the other hand, when the Aβ aggregates become available, the signaling through the *Wnt *pathway might be affected: GSK-3β activates, β-catenin destroyed, and the *Wnt *mediated gene transcription is stopped (Right Panel). Several potential mechanisms of how Aβ aggregates affect *Wnt *signaling might be possible: (a) Aβ may bind to the *Wnt *ligand (scavenger effect), (b) Aβ may directly interact with the Fz receptor, (c) Dkk-1 may become available and block the transduction at the receptor level, or (d) Aβ may affect calcium flux by direct activation of the α7-nicotinic ACh and/or NMDA receptors. As a consequence, GSK-3β is activated and β-catenin function attenuated.

At the beginning of this decade (Early in 2000), we found a relationship between a loss of the *Wnt *signaling pathway activity and AD. Early studies in our laboratory suggested a relationship between Aβ-induced neurotoxicity and an impairment of this signaling pathway, Figure 1[4,24-26]. Several independent studies are consistent with the idea that *Wnt *signaling components are altered in AD [27-33]. As a result, we have studied whether or not the activation of the *Wnt *signaling pathway may be used as a therapeutic strategy to treat AD.

### The activation of the *Wnt *Signaling Pathway Prevents Aβ-induced Neurotoxicity

A considerable amount of data has led to a quest to understand the role *Wnt *signaling may play in AD. β-catenin levels are markedly reduced in AD patients carrying presenilin-1 (PS-1) inherited mutations [29]. In fact, several studies have shown that familial AD-linked PS-1 proteins form multi-protein complexes with β-catenin and GSK-3β [34-36]. Early studies in our laboratory suggested a relationship between Aβ-induced neurotoxicity and lower cytoplasmic levels of β-catenin. Inhibition of GSK-3β by lithium was shown to protect rat hippocampal neurons from Aβ-induced damage [25,26,37]. These evidences led us to propose that a sustained loss of *Wnt *signaling function could be involved in the Aβ dependent neurodegeneration observed in AD [24,37].

The enzyme GSK-3β, a key modulator in the *Wnt *canonical, has its activity related with the neuropathology present in AD. GSK-3β is widely expressed throughout the rat CNS [38], with particularly high levels of expression in the hippocampus. In cultured hippocampal neurons, it is expressed throughout the cell bodies, including dendritic spines [39]. The presence of GSK-3β within dendrites and dendritic spines suggests that it may have a role in synaptic function. Recently, Collingridge and coworkers obtained evidence for a role of GSK-3β in NMDA receptor-dependent long-term depression (LTD) at CA3-CA1 synapses of 2-week-old rats. They found that a variety of inhibitors of GSK-3β were able to prevent the induction of LTD when loaded into the recorded neuron using a patch pipette. These structurally unrelated inhibitors, SB415286, lithium and kenpaullone, prevented the induction of LTD over the appropriate concentration range at which they inhibited GSK-3β [40]. Previous studies have shown that following the induction of LTP there is inhibition of GSK-3β activity [41]. In summary, GSK-3β is required for LTP and provides a mechanism by which LTP can inhibit LTD, therefore the regulation of GSK-3β activity provides a mechanism to preserve information encoded during LTP from erasure by subsequent LTD. Whether or not these functions or the deregulation of these functions are important early or late features in the development of neurodegenerative diseases remains to be determined [39].

**Figure 2:**
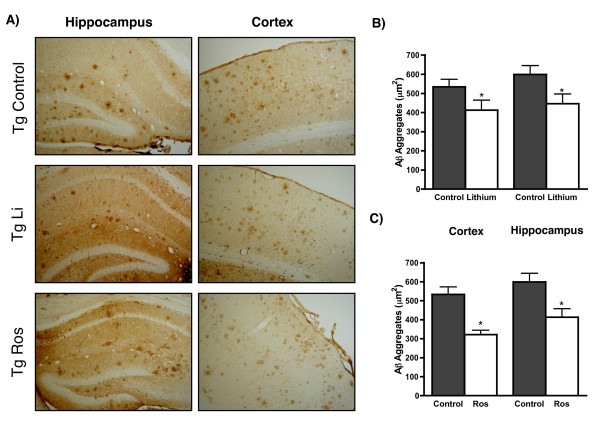
**Treatments with Lithium and Rosiglitazone reduce the amount of total Aβ in brains of APPswe+PSEN1ΔE9 mice**. **(A) **Hippocampal and cortical slices from transgenic mice APPswe+PSEN1ΔE9 (Tg) stained against Aβ. Photos are of Tg control and Tg treated animals with Lithium or Rosiglitazone. Figures **B **and **C **show the average Aβ plaque area (μm^2^) in the hippocampus and cortex after the treatments of lithium **(B) **and rosiglitazone **(C) **respectively, with bars representing the average plaque area for each specific treatment ± S.E (n = 5). Asterisks indicate significant differences, p < 0.05.

In AD brain, active GSK-3β (also known as tau kinase I) is mainly found in neuronal cell bodies and neurites [42], where it is found co-localized with the neurofibrillary changes observed in AD brains. The activation of the enzyme GSK-3β, the hyperphosphorylation of tau protein, and the loss of the microtubular network have all been observed in primary cultures of rat hippocampal and human cortical neurons exposed to the Aβ peptide [43,44]. Interestingly, it has been observed that blocking GSK-3β activity prevents tau hyper-phosphorylation and promotes its binding to the microtubular network [45]. Lithium, which has long been used to treat bipolar disorders [46], has been shown to be a competitive inhibitor of GSK-3 with respect to magnesium, a property not found in other group I metal ions [47]. This may account for its ability to act as a mood-stabilizing drug [48], though other actions of lithium, such as its well-known ability to inhibit inositol-1,4 bis-phosphate 1-phosphatase and inositol-1(or 4)-mono-phosphatase, could also explain or contribute to its therapeutic effects [49]. Studies from different laboratories indicated that lithium protects rat hippocampal neurons from Aβ insults suggesting that a sustained loss of the *Wnt *signaling function may be involved in the Aβ dependent neurodegeneration observed in AD [26,27]. Furthermore, recent evidence suggests that lithium is neuro-protective against a variety of neurodegenerative conditions [46,50], and it is noteworthy that lithium reduces the prevalence of AD in elderly patients with bipolar disorder [51]. Ongoing clinical trials are evaluating the efficacy of this drug to lower tau and β-amyloid levels in the cerebral spinal fluid of AD patients . Recent studies in our laboratory, using double transgenic mice (APP_*SWE *_+ PSEN1ΔE9) indicated that lithium injection prevents the behavioral disturbances of the animals, reducing the size of the amyloid plaque, Figure 2A, B (Toledo & Inestrosa, unpublished results).

The exposure of rat hippocampal neurons to Aβ result in three hallmarks related with *Wnt *signaling: (a) destabilization of endogenous levels of β-catenin, (b) an increase in GSK-3β activity and (c) a decrease in *Wnt *target gene transcription. *In vitro *studies have shown that the activation of the canonical *Wnt *signaling pathway by *Wnt-3a *and *Wnt-7a *conditioned media were able to overcome the neurotoxic consequences induced by Aβ [52,53]. Moreover the exposure of neurons in culture to Aβ induces apoptosis and promotes tau hyperphosphorylation through GSK-3β activity [54,55].

The Wnt/Ca^2+ ^pathway signals through Dvl to induce calcium influx and the activation of protein kinase C (PKC) [56]. The inactivation of GSK-3β by PKC, leads to two main consequences: reduced phosphorylation of tau protein and reduced degradation and subsequent accumulation of cytoplasmic β-catenin [25,53]. PKC isoenzymes are degraded in a differential manner upon Aβ exposure. The modulation of PKC affects Aβ neurotoxicity, as the activation of this enzyme by phorbol-12-myristate 13-acetate increases cell viability of rat hippocampal neurons and neuroprotection towards Aβ. PKC inhibits GSK-3β through serine 9 phosphorylation preventing the cytoplasmic β-catenin degradation and thus, activating the transcription of *Wnt *target genes such as engrailed and cyclin-D1. *Wnt*-3a and lithium mimicked PKC activation [25,53]. The regulation of some components of the *Wnt *signaling pathway by Ca^2+^-dependent PKC iso-forms, may be important in controlling the neurotoxic process induced by Aβ. As a result, the activation of the *Wnt *signaling pathway has been proposed [25,26,52,53,57] as a therapeutic target for the treatment of AD.

The loss of *Wnt *signaling cannot be just attributed to a loss of function. The reduction in the Wnt signaling can be due to a gain of function of inhibitors of the Wnt signaling. Studies by Caricasole et al (2004) [30] showed that the exposure of cortical neurons to Aβ induced the expression of the secreted glycoprotein Dkk-1. Dkk-1 negatively modulates the canonical Wnt signaling pathway, thus activating the tau-phosphorylating enzyme GSK-3β [58]. Dkk-1 was induced at later times after Aβ exposure, and its expression was dependent on the tumor suppressing protein p53. The antisense induced knockdown of Dkk-1 attenuates the reduction in the phosphorylated (inhibited) form of GSK-3β, and a selective GSK-3β inhibitor prevents tau hyperphosphorylation in neurons challenged with Aβ. The Dkk-1 knockdown also attenuates neuronal apoptosis. These mechanisms may be relevant to the AD pathology because Dkk-1, which is hardly found in the healthy brain, is highly expressed in the AD brain where it is found around the amyloid plaques and co-localizes with neurofibrillary tangles and dystrophic neurites. These studies indicate that induction of Dkk-1 contributes to the pathological cascade triggered by Aβ and is critically involved in the process of tau-phosphorylation. These results strengthen the hypothesis that an impairment of the *Wnt *pathway contributes to the pathophysiology of AD [4,5,24,58,59]

Another mechanism for the inhibition of the *Wnt *signaling is by sFRPs, which are also capable to disrupt the *Wnt *network signaling. Increased expression of sFRP 1, 2, 3 and 5 has been reported in Inherited Retinal Degenerations such as *Retinitis Pigmentosa*, which is characterized by progressive loss of photoreceptors due to apoptosis [60,61]. sFRPs can regulate apoptosis *in vitro*, in fact, sFRP2 a member of the family of secreted Frizzled-related proteins [62], is also known as secreted apoptosis-related protein-1 (SARP-1) [63]. They appear to interact with the *Wnt*/β-catenin or *Wnt*/Frizzled signaling pathway, which includes routes to apoptotic activation. As discussed by Baranski et al (2000) [64] it is also possible that sFRPs operate agonistically to *Wnt *signaling in some circumstances: for example, sFRP2 (SARP-1) increases resistance of MCF7 breast adeno-carcinoma cells to apoptotic signals, whereas sFRP1 (SARP-2) sensitizes the same cell [63] via opposing effects on intracellular β-catenin levels. These results suggest that intercellular signals via the *Wnt *pathways are substantially disrupted in the degenerative state, and that targeting of sFRPs to key areas of the neuro-retina may mediate mechanisms promoting or antagonizing cell death, similar mechanisms may also be true for neurodegenerative diseases such as AD.

Genetic epidemiological data show a link between *Wnt *signaling and AD. The analysis of single-nucleotide polymorphisms show an increased risk for AD in populations with inheritance of the apo-lipoprotein E-ε4 (APOE-ε4) allele, including both sporadic and late-onset familial forms of the disease [65]. Recently, it was reported that APOE-ε4 causes the inhibition of the canonical *Wnt *signaling pathway in PC12 cells upon stimulation with *Wnt-7a *as determined by luciferase activities and nuclear β-catenin levels [66]. Epidemiological studies also estimates that 42–48% of AD patients do not present the APOE-ε4 allele, suggesting that additional genetic or environmental factors could play essential roles in the disease [67]. Genome-wide screens have identified several regions that show significant linkage to AD. The reported linkage peaks of chromosome 12 show significant association with AD, particularly one region located in the vicinity of the LRP 6 [32]. Since LRP5/6 encodes a co-receptor for the *Wnt *pathway, its association with AD was studied. Results unveil an association between a highly conserved coding sequence LRP 6 polymorphism (Ile1062Val) and the risk to develop late-onset AD in APOE-ε4 allele carriers. Interestingly, the Val 1062 variant of LRP 6 causes a reduced activation of a β-catenin-responsive reporter gene in HEK293T/STF recombinant cells [32], suggesting that a reduced efficiency of the canonical *Wnt *signaling pathway may predispose people to AD.

Accumulation of cytoplasmic inclusion bodies in many neurodegenerative diseases, including AD, might result from dysfunction of the ubiquitin-proteasome system [68,69]. This system degrades many cellular proteins, including β-catenin. *Wnt *signaling activation causes the dissociation of the multiprotein complex that contains, among others, GSK-3β and β-catenin. This prevents GSK-3β from phosphorylating β-catenin [70]. Un-phosphorylated β-catenin becomes resistant to proteosomal degradation [71] and moves to the nucleus, where it regulates gene expression after interacting with members of the TCF/LEF family of transcription factors. Genes that are affected by the canonical *Wnt *pathway are involved in the regulation of neuronal survival and homeostasis (such Bcl-2, α7-nicotinic AChR, insulin degrading enzyme, CaMKIV and neuroligin) [72-76]. Phosphorylation of β-catenin labels it for ubiquitination and rapid proteasomal degradation. Studies by Ghanevati and Miller (2005) [31] indicated that phospho-β-catenin accumulated as detergent-insoluble, punctuate cytoplasmic inclusions in hippocampal pyramidal neurons more abundantly in AD brain than in aged controls. Phospho-β-catenin is partially sequestered within granulo-vacuolar degeneration bodies but not lysosomes, indicating sequestration within autophagosomes. Exposure of neuronal cultures to proteasome inhibitors induced formation of detergent-insoluble, phospho-β-catenin-positive cytoplasmic inclusions that coalesced into aggresomes and colocalized with γ-tubulin and vimentin. These aggregates were associated with apoptotic cell death and with activation of caspase-3, c-Jun-N-terminal kinases, and c-Jun [31]. These findings suggest that the accumulation of phospho-β-catenin in AD result from impaired proteasomal function. Recently, it was found that the up-regulation of β-catenin during tau-hyperphosphorylation prevents neuronal cells from going into apoptosis. Furthermore, increasing levels of hyperphosphorylated tau was correlated with diminished levels of phospho-β-catenin and increased levels of nuclear β-catenin. Moreover, the knockdown of β-catenin increases the number of apoptotic cells and antagonizes the anti-apoptotic effects of tau [77]. These results support the role of β-catenin and therefore the Wnt/β-catenin signaling in neuronal survival following Aβ insult in AD.

In mammals, Fz genes have been implicated in a variety of developmental processes, including axonal outgrowth and guidance in the central nervous system [78,79], the survival of cerebellar neurons [80], hippocampal and visuospatial learning [81], and the control of the neural tube closure [82]. Rat Fz1 and Fz2 have been studied in greatest detail and provide the best discrimination of the *Wnt *pathways, referred to as *Wnt*/β-catenin pathway [83,84], versus the *Wnt-*Ca^2+ ^pathway [23,85]. An exhaustive study of the possible associations between the known 19 *Wnt *ligands and the 10 Fz has not been carried out, although some combinations seem to convey a meaningful intracellular signal [86,87], including human Fz1 and *Wnt-3a *[88], and Fz5 and *Wnt-7a *[89]. Although *Wnt *signaling pathway and Fz receptors have been shown to participate in the development and maintenance of the nervous system, little is known about the expression of Fz in the mammalian brain. Through the analysis of *in-situ *hybridization of adult mice brains, it was found that numerous Fz receptors and *Wnt *ligands are expressed across the brain [90]. Knowledge of the pattern of expression of Fz receptors and *Wnt *ligands, may contribute to the understanding of the *in vivo Wnt *signaling in the adult brain. More recently, a high-throughput methodology that allows the analysis of expression of 20000 genes, revealed that the adult brain of mice expresses different components of the *Wnt *signaling pathway [91].

The activation of the canonical *Wnt *signaling pathway protects hippocampal neurons against the toxicity of Alzheimer's Aβ, however, the role played by the *Wnt *receptors Fz has not been studied. Recently we found that Fz1 mediates the activation of the canonical *Wnt*/β-catenin pathway by *Wnt-3a *in PC12 cells. In addition, the protective effect of *Wnt-3a *against the toxicity of Aβ oligomers was modulated by Fz1 expression levels. Over-expression of Fz1 significantly increased cell survival induced by *Wnt-3a *and diminished caspase-3 activation and β-catenin degradation, these *Wnt-3a *effects are potentiated by over-expression of Fz1, but not Fz2, and are significantly reduced when Fz1 is knocked down by antisense oligonucleotides in PC12 cells [92]. Over-expression of wild-type β-catenin, but not a transcriptionally inactive mutated version, prevented the toxicity of Aβ suggesting that the transcription of *Wnt *target genes may be involved in these events. This was confirmed by co-transfecting both Fz1 and the inactive form of β-catenin, which did not elicit protection levels similar to those shown with endogenous β-catenin. Fz1 is expressed in the adult rat hippocampus and cortex, and in cultured hippocampal neurons where *Wnt-3a *also protects against Aβ toxicity, an effect that was decreased by knocking-down Fz1 expression [92]. The neuro-protective effect of *Wnt-3a *modulated by Fz1 expression suggests that the activation of the canonical *Wnt *signaling pathway prevents the neurotoxicity induced by the Aβ peptide and again suggest a therapeutic potential for this signaling pathway in the treatment of AD.

The signal transduction mechanisms involved in Aβ-induced neuronal dysfunction remain to be fully understood; the identity of the protein receptor(s) involved in neuronal Aβ binding has not been identified. Studies by Ferreira and coworkers in Brazil [93], have identified a number of peptides that bind Aβ and are homologous to neuronal receptors putatively involved in Aβ interactions, using phage display of peptide libraries [33,94]. Through this methodology they have found an heptapeptide called IQ, which is common to nAChRs with the ability to bind Aβ with a nanomolar affinity [94]. This binding is enough to block the inhibition of nAChRs by Aβ when it was studied in PC12 cells. These results demonstrate that a region found in nAChRs acts as a receptor to Aβ and allow us to hypothesize the role of nAChRs as receptors of Aβ in the CNS. More recently, Ferreira and coworkers reported a cysteine-linked cyclic heptapeptide (denominated cSP) that is highly homologous to the extracellular cysteine-rich domain (CRD) of several members of the Fz family of *Wnt *receptors. Based on this homology, they investigated the interaction between Aβ and Fz, and found that Aβ binds to the Fz CRD at or in close proximity to the *Wnt *binding site and inhibits β-catenin accumulation, nuclear translocation and *Wnt*-targeted gene transcription [33]. Interestingly, the cSP peptide completely blocks Aβ binding to Fz and prevents inhibition of the *Wnt *signaling cascade. These results indicate that the Aβ binding site in Fz is homologous to cSP and that this is a relevant target for Aβ neurotoxicity. Furthermore, they suggest that blocking the interaction of Aβ with Fz might lead to novel therapeutic approaches to prevent neuronal dysfunction in AD.

### Cross-talk of different signaling pathways with the Wnt Pathway leads to neuroprotection against Aβ Neurotoxicity

The emerging role of *Wnt *signaling as a therapeutic target for treatment of AD led us to evaluate potential pathways that interact with the *Wnt *signaling:

**(a) **Cholinergic dysfunction has been observed in AD patients, indicating its relationship with Aβ neurotoxicity. Degenerated pre-synaptic cholinergic neurons that ascend from the basal forebrain to cortical and hippocampal areas have been observed [95]. In relation to AD, it is well known that M1 agonists increase the non-amyloidogenic processing of the amyloid precursor protein (APP), reducing Aβ production [96] and tau phosphorylation [97]. In addition, M1 muscarinic receptor activation by the specific agonist AF267B induces the phosphorylation/inactivation of GSK-3β in cortical neuronal cultures from transgenic mice that overexpress GSK-3β. Aβ treatment, as well as transgenic mice that over-express GSK-3β, shows decreased levels of Ser-9 phosphorylation, thus GSK-3β is activated. On the contrary, M1 agonist treatments decrease GSK-3β activity. In this manner, Ser-9 phosphorylation/inactivation of GSK-3β by M1 mAChR stimulation is probably mediated by a mechanism that involves protein kinase C (PKC), since a PKC inhibitor blocked M1 muscarinic receptor activation-induced Ser-9 phosphorylation. Interestingly, it has been shown that PKC protects from apoptosis induced by Aβ [23,98]. Hippocampal neurons exposed to Aβ toxicity induced the activation of GSK-3β, which was prevented by the activation of M1 muscarinic receptor. The protection observed *in vitro *was later found *in vivo*; chronic treatment with the specific M1 agonist AF267B, improved the spatial memory and reduced the Aβ load in the hippocampus of a triple transgenic mouse [99]. Thus, the M1 muscarinic activation and the *Wnt *signaling pathway interact, leading to potential neuroprotection against Aβ toxicity.

**(b) **The use of non-steroidal anti-inflammatory drugs (NSAIDs) has been observed to reduce the risk for AD [100]. The NSAIDs have been proposed to act by inhibiting the secretases that cleave the APP in the amyloidogenic pathway to render Aβ. Moreover, NSAIDs dramatically reduce the secretion of Aβ_1–42 _in cells *in vitro *[101-104]. A bi-functional compound that includes Ibuprofen (an anti-inflammatory drug) and prostigmine (a cholinesterase inhibitor), IBU-PO protects hippocampal neurons from Aβ neurotoxicity, increases the viability of Aβ-challenged hippocampal neurons, and enhances neurite growth [105]. The protection observed is the result of the *Wnt *signaling activation, since the increase in the activity of GSK-3β induced by Aβ is down-regulated by co-treatment with IBU-PO. In addition, this down-regulation occurs through induction of Ser-9 phosphorylation. Transgenic mice that over-express GSK-3β show low levels of Ser-9 phosphorylation and the IBU-PO treatment induces an increase in this phosphorylation [105]. Compounds such as IBU-PO, which mimic the activation of the *Wnt *signaling pathway, could eventually rescue neurons from cytotoxicity through GSK-3β inhibition, which may be of potential benefit for the treatment of AD patients.

**(c) **Treatment with some antioxidants has been suggested as an avenue for the treatment of AD [106]. We have studied whether or not some antioxidants are able to affect the canonical *Wnt *signaling pathway. Treatments with Trolox (an hydro-soluble analogue of vitamin E) and 17β-estradiol, but not vitamin C, increases the cytoplasmic levels of β-catenin and inhibits the increase in GSK-3β activity observed when neurons are exposed to Aβ [52]. In this context, we ask whether or not the activation of the *Wnt *signaling by anti-oxidant treatment increases the mRNA levels of some of the components of the Wnt pathway. Results indicated that both *Wnt-7a *and *Wnt-5a *ligands were induced by the anti-oxidant treatment [52]. A similar effect was observed for engrailed-1 mRNA. In this context, it is interesting to mention that at least 4 *Wnt *ligands (*Wnts *4, 11, 5a and 7a) present in embryonic hippocampal neurons are also expressed in the adult rat brain [107], indicating that at least some of the *Wnt *ligands are present throughout the entire lifespan of mammals.

**(d) **The relative importance of hydrogen peroxide and free radicals in the neurodegenerative processes triggered by Aβ had been previously addressed [106]. For example, Schubert and coworkers [108], demonstrated that the addition of catalase (an enzyme that inactivates hydrogen peroxide) to neuronal cultures exposed to Aβ, results in the prevention of neurodegenerative changes. More recently, we found that proliferation of peroxisomes, intracellular organelles that destroy the excess of cellular hydrogen peroxide, also prevent the neurotoxic effects generated by Aβ in rat hippocampal cells [109]. The drugs used to trigger the peroxisomal proliferation normally activate a member of a family of nuclear receptors known as peroxisome proliferator activated receptors (PPAR), particularly the PPARα. Such drugs increase β-catenin content in hippocampal neurons, suggesting an interaction with the *Wnt *signaling pathway [109].

Another PPAR, known as the PPARγ, plays an important role in the regulation of lipid metabolism [110]. In addition, PPARγ is the target of the insulin-sensitizing thiazolidinediones (TZDs) drugs, used to treat type II diabetes. Recent studies suggest that treatment of insulin resistance with a PPARγ agonist retards the development of AD [111], and TZDs have been proposed as potential therapeutic agents for both diabetes and AD [112]. Most of the neuroprotective effects of TZDs are ascribed to either improved insulin sensitivity or to their anti-inflammatory action through PPARγ activation in reactive astrocytes and microglia [113,114]. Studies in our laboratory, demonstrated that the activation of PPARγ by three different TZDs was able to prevent the neurodegeneration induced by the Aβ. The activation with the PPARγ agonists modulate *Wnt *signaling components, including the inhibition of GSK-3β activity, the increase in both the cytoplasmic and nuclear levels of β-catenin, as well as the transcription of *Wnt *target genes *en-1 *and *cyclin-D1 *[115]. Previous studies in our laboratory indicated that Bcl-2 may be a *Wnt *target gene [72,116]. Recent studies with neurons containing high and low PPARγ levels, suggest that Bcl-2 plays a key role in the neuroprotection to both hydrogen peroxide and Aβ [72]. In fact, NGF-differentiated PC12 neuronal cells that over-express PPARγ are resistant to Aβ-induced apoptosis and to ROS increase after exposure to hydrogen peroxide. Conversely, cells expressing a dominant negative mutant of PPARγ show increased Aβ-induced apoptosis and alterations by oxidative stress. Neurons over-expressing PPARγ show a 4.5-fold increase in Bcl-2 content, whereas in dominant negative PPARγ-expressing cells, Bcl-2 is barely detected. Bcl-2 knockdown by siRNA in neurons over-expressing PPARγ results in increased sensitivity to Aβ and oxidative stress. Finally, PPARγ pro-survival action is independent of the signal regulated MAPK or the Akt pathways [72]. These results suggest that PPARγ supports neuronal survival by a mechanism that involves an increased expression of Bcl-2. An alternative mechanism that could protect neurons from the Aβ toxicity has been proposed. In fact, TZD, an agonist of PPARγ, induced an increase in the clearance mechanism of the Aβ peptide [117]. Interestingly, the activation of PPARγ by rosiglitazone improves learning and memory in a mouse model of AD, together with a reduction in Aβ in the brains of Tg mice [118]. Recent studies in our laboratory, using the APP_SWE _+ PSEN1ΔE9 double transgenic mice, indicated that rosiglitazone administration prevents the behavioral and the inflammatory-glial disturbances observed in transgenic animals, thus reducing amyloid plaque size, Figure 2A,C (Toledo & Inestrosa, unpublished results).

**Figure 3:**
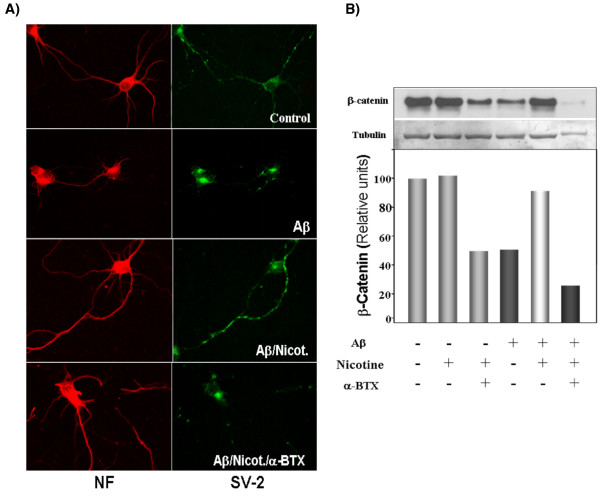
**Activation of α7nAChR with nicotine protects hippocampal neurons from Aβ fibers**. **(A) **Hippocampal neurons (10 DIV) were expose to Aβ_1–40 _fibers 5 mM for 6 h in the absence or presence of nicotine 10 μM. The immunostainings of the protein neurofilament (NF) shows the loss of dendrites, and the presynaptic protein SV-2, show a significant reduction upon exposure to Aβ. Nicotine is able to overturn the damage cause by Aβ. This reversion is specific to the nicotinic receptor since this protective effect was blocked by α-bungarotoxin (100 nM). **(B) **These effects are observed by western-blot of the total levels of total β-catenin, in which the reduction of β-catenin is prevented by nicotine and blocked with α-BTX.

**(e) **Recently, we reported that *Wnt-7a *induces dissociation of the APC protein from the α-catenin cytoplasmic complex and the interaction of APC with the α7-nAChR in hippocampal neurons. In the CNS, α7-nAChRs are involved in several aspects of brain function, affecting neuronal development [119], learning, and memory [120]. Because of their high permeability to calcium ions, α7-nAChRs influence synaptic efficacy and induction of LTP [121]. In Parkinson's and AD, a decrease in the amount of α7-nAChRs has been found [122,123]. *Wnt-7a *is able to induce the re-localization of APC to membranes, clustering of APC in neurites, and co-clustering of APC with the presynaptic protein markers, including P-synapsin, SV2, and synaptotagmin. Moreover, *Wnt-7a *also increases the number and size of co-clusters of α7-nAChRs and APC in pre-synaptic nerve terminals [73]. These short-term changes in α7-nAChRs take place within a few minutes after ligand exposure and involve translocation to the plasma membrane without affecting total levels of the receptor. Long-term exposure to *Wnt-7a *increases both nAChR α7 subunit levels in an APC independent manner and clusters of α7-nAChRs in neurites via an APC dependent process [73]. These results suggest that α7-nAChR could be a target of the *Wnt *pathway by regulating the pre-synaptic localization of APC and α7-nAChRs, with APC serving as an intermediary in the α7-nAChR re-localization process. Activation of α7-nAChR with nicotine protects culture of hippocampal neurons from Aβ aggregates and this protective effect of nicotine was blocked by α-bungarotoxin. These effects are observed at the immunofluorescence level, as well as at the level of β-catenin by western blot (Figure 3).

Modulation by *Wnt *signaling may be essential for α7-nAChR expression and function in synapses. Perhaps therapies aimed to activate *Wnt *signaling could be effective in treatment of AD, especially if they prevent loss of α7-nAChRs from synaptic regions, as well as of other important synaptic proteins. These compounds and new ones yet to be discovered, which inhibit the GSK-3β activity and/or enhance *Wnt *signaling, could lead to the reduction of neuropathological factors involved in AD. The crosstalk of the *Wnt *signaling pathway with other cellular pathways is opening new possibilities for therapy.

## Conclusion

Several lines of evidence indicate that deregulated *Wnt *signaling may play a role in the pathogenesis of AD. The potential use of GSK-3β as a clinical target in AD has been discussed, including the activation of M1 muscarinic receptor and PKC, the use of anti-inflammatory-ChE bifunctional compounds, PPAR agonists, and some antioxidants, all of which may play a role by regulating the *Wnt*/β-catenin signaling. So far the mechanisms by which extracellular Aβ causes its different intra-neuronal effects have not been clarified. *Wnt-7a *signaling stimulates clustering of pre-synaptic proteins and modulates the synaptic vesicle cycle by inducing recycling and exocytosis of synaptic vesicles. Aβ oligomers bind to the central synapse at the postsynaptic region, and we have found that *Wnt-5a *plays an attenuating role in Aβ neurotoxicity. In addition, this ligand modulates the insertion of glutamate receptors in the postsynaptic region of synapses. All of these data opens a novel therapeutic window in AD treatment.

## Competing interests

The authors declare that they have no competing interests.

## Authors' contributions

NCI participated in the design of the review and writing of the manuscript. EMT performed some experiments presented, and contribute to the writing and revision of the manuscript. Both authors read and approved the final manuscript.

## References

[B1] Selkoe DJ (2002). Alzheimer's disease is a synaptic failure. Science.

[B2] Logan CY, Nusse R (2004). The *Wnt* signaling pathway in development and disease. Annu Rev Cell Dev Biol.

[B3] Nusse R (2008). *Wnt* signaling and stem cell control. Cell Res.

[B4] Inestrosa N, De Ferrari GV, Garrido JL, Alvarez A, Olivares GH, Barria MI, Bronfman M, Chacon MA (2002). *Wnt* signaling involvement in β-amyloid-dependent neurodegeneration. Neurochem Int.

[B5] Caricasole A, Copani A, Caruso A, Caraci F, Iacovelli L, Sortino MA, Terstappen GC, Nicoletti F (2003). The *Wnt* pathway, cell-cycle activation and β-amyloid: novel therapeutic strategies in Alzheimer's disease?. Trends Pharmacol Sci.

[B6] Moon RT, Kohn AD, De Ferrari GV, Kaykas A (2004). *WNT* and β-catenin signalling: diseases and therapies. Nat Rev Genet.

[B7] Gordon MD, Nusse R (2006). *Wnt* signaling: multiple pathways, multiple receptors, and multiple transcription factors. J Biol Chem.

[B8] Mikels AJ, Nusse R (2006). Wnts as ligands: processing, secretion and reception. Oncogene.

[B9] Dale TC (1998). Signal transduction by the *Wnt* family of ligands. Biochem J.

[B10] Niehrs C (2006). Function and biological roles of the Dickkopf family of *Wnt* modulators. Oncogene.

[B11] Diep DB, Hoen N, Backman M, Machon O, Krauss S (2004). Characterisation of the *Wnt* antagonists and their response to conditionally activated *Wnt* signalling in the developing mouse forebrain. Brain Res Dev Brain Res.

[B12] Mao B, Wu W, Li Y, Hoppe D, Stannek P, Glinka A, Niehrs C (2001). LDL-receptor-related protein 6 is a receptor for Dickkopf proteins. Nature.

[B13] Perrimon N, Bernfield M (2000). Specificities of heparan sulphate proteoglycans in developmental processes. Nature.

[B14] Binari RC, Staveley BE, Johnson WA, Godavarti R, Sasisekharan R, Manoukian AS (1997). Genetic evidence that heparin-like glycosaminoglycans are involved in wingless signaling. Development.

[B15] Lin X, Perrimon N (1999). Dally cooperates with Drosophila Frizzled 2 to transduce Wingless signalling. Nature.

[B16] Baeg GH, Lin X, Khare N, Baumgartner S, Perrimon N (2001). Heparan sulfate proteoglycans are critical for the organization of the extracellular distribution of Wingless. Development.

[B17] Colombres M, Henriquez JP, Reig GF, Scheu J, Calderon R, Alvarez A, Brandan E, Inestrosa NC (2008). Heparin activates *Wnt* signaling for neuronal morphogenesis. J Cell Physiol.

[B18] Moon RT, DeMarais A, Olson DJ (1993). Responses to *Wnt* signals in vertebrate embryos may involve changes in cell adhesion and cell movement. J Cell Sci Suppl.

[B19] Du SJ, Purcell SM, Christian JL, McGrew LL, Moon RT (1995). Identification of distinct classes and functional domains of Wnts through expression of wild-type and chimeric proteins in Xenopus embryos. Mol Cell Biol.

[B20] Mikels AJ, Nusse R (2006). Purified *Wnt5a* protein activates or inhibits β-catenin-TCF signaling depending on receptor context. PLoS Biol.

[B21] Kohn AD, Moon RT (2005). *Wnt* and calcium signaling: β-catenin-independent pathways. Cell Calcium.

[B22] Miller JR, Hocking AM, Brown JD, Moon RT (1999). Mechanism and function of signal transduction by the *Wnt*/β-catenin and *Wnt*/Ca2+ pathways. Oncogene.

[B23] Kuhl M, Sheldahl LC, Park M, Miller JR, Moon RT (2000). The *Wnt*/Ca2+ pathway: a new vertebrate *Wnt* signaling pathway takes shape. Trends Genet.

[B24] De Ferrari GV, Inestrosa NC (2000). *Wnt* signaling function in Alzheimer's disease. Brain Res Brain Res Rev.

[B25] Garrido JL, Godoy JA, Alvarez A, Bronfman M, Inestrosa NC (2002). Protein kinase C inhibits amyloid β peptide neurotoxicity by acting on members of the *Wnt* pathway. FASEB J.

[B26] De Ferrari GV, Chacon MA, Barria MI, Garrido JL, Godoy JA, Olivares G, Reyes AE, Alvarez A, Bronfman M, Inestrosa NC (2003). Activation of *Wnt* signaling rescues neurodegeneration and behavioral impairments induced by β-amyloid fibrils. Mol Psychiatry.

[B27] Alvarez G, Munoz-Montano JR, Satrustegui J, Avila J, Bogonez E, Diaz-Nido J (2002). Regulation of tau phosphorylation and protection against β-amyloid-induced neurodegeneration by lithium. Possible implications for Alzheimer's disease. Bipolar Disord.

[B28] Takashima A, Murayama M, Murayama O, Kohno T, Honda T, Yasutake K, Nihonmatsu N, Mercken M, Yamaguchi H, Sugihara S, Wolozin B (1998). Presenilin 1 associates with glycogen synthase kinase-3β and its substrate tau. Proc Natl Acad Sci USA.

[B29] Zhang Z, Hartmann H, Do VM, Abramowski D, Sturchler-Pierrat C, Staufenbiel M, Sommer B, Wetering M van de, Clevers H, Saftig P (1998). Destabilization of β-catenin by mutations in presenilin-1 potentiates neuronal apoptosis. Nature.

[B30] Caricasole A, Copani A, Caraci F, Aronica E, Rozemuller AJ, Caruso A, Storto M, Gaviraghi G, Terstappen GC, Nicoletti F (2004). Induction of Dickkopf-1, a negative modulator of the *Wnt* pathway, is associated with neuronal degeneration in Alzheimer's brain. J Neurosci.

[B31] Ghanevati M, Miller CA (2005). Phospho-β-Catenin Accumulation in Alzheimer's Disease and in Aggresomes Attributable to Proteasome Dysfunction. J Mol Neurosci.

[B32] De Ferrari GV, Papassotiropoulos A, Biechele T, Wavrant De-Vrieze F, Avila ME, Major MB, Myers A, Saez K, Henriquez JP, Zhao A (2007). Common genetic variation within the low-density lipoprotein receptor-related protein 6 and late-onset Alzheimer's disease. Proc Natl Acad Sci USA.

[B33] Magdesian MH, Carvalho MM, Mendes FA, Saraiva LM, Juliano MA, Juliano L, Garcia-Abreu J, Ferreira ST (2008). Amyloid-β binds to the extracellular cysteine-rich domain of Frizzled and inhibits *Wnt*/β-catenin signaling. J Biol Chem.

[B34] Yu G, Chen F, Levesque G, Nishimura M, Zhang DM, Levesque L, Rogaeva E, Xu D, Liang Y, Duthie M (1998). The presenilin 1 protein is a component of a high molecular weight intracellular complex that contains β-catenin. J Biol Chem.

[B35] Nishimura M, Yu G, Levesque G, Zhang DM, Ruel L, Chen F, Milman P, Holmes E, Liang Y, Kawarai T (1999). Presenilin mutations associated with Alzheimer disease cause defective intracellular trafficking of β-catenin, a component of the presenilin protein complex. Nat Med.

[B36] Kang DE, Soriano S, Frosch MP, Collins T, Naruse S, Sisodia SS, Leibowitz G, Levine F, Koo EH (1999). Presenilin 1 facilitates the constitutive turnover of β-catenin: differential activity of Alzheimer's disease-linked PS1 mutants in the β-catenin-signaling pathway. J Neurosci.

[B37] Inestrosa NC, Alvarez A, Godoy J, Reyes A, De Ferrari GV (2000). Acetylcholinesterase-amyloid-β-peptide interaction and *Wnt* signaling involvement in Aβ neurotoxicity. Acta Neurol Scand Suppl.

[B38] Leroy K, Brion JP (1999). Developmental expression and localization of glycogen synthase kinase-3β in rat brain. J Chem Neuroanat.

[B39] Peineau S, Bradley C, Taghibiglou C, Doherty A, Bortolotto ZA, Wang YT, Collingridge GL (2008). The role of GSK-3 in synaptic plasticity. Br J Pharmacol.

[B40] Peineau S, Taghibiglou C, Bradley C, Wong TP, Liu L, Lu J, Lo E, Wu D, Saule E, Bouschet T (2007). LTP inhibits LTD in the hippocampus via regulation of GSK3β. Neuron.

[B41] Hooper C, Markevich V, Plattner F, Killick R, Schofield E, Engel T, Hernandez F, Anderton B, Rosenblum K, Bliss T (2007). Glycogen synthase kinase-3 inhibition is integral to long-term potentiation. Eur J Neurosci.

[B42] Takahashi M, Tomizawa K, Kato R, Sato K, Uchida T, Fujita SC, Imahori K (1994). Localization and developmental changes of tau protein kinase I/glycogen synthase kinase-3 β in rat brain. J Neurochem.

[B43] Bhat RV, Budd Haeberlein SL, Avila J (2004). Glycogen synthase kinase 3: a drug target for CNS therapies. J Neurochem.

[B44] Plattner F, Angelo M, Giese KP (2006). The roles of cyclin-dependent kinase 5 and glycogen synthase kinase 3 in tau hyperphosphorylation. J Biol Chem.

[B45] Avila J, Lucas JJ, Perez M, Hernandez F (2004). Role of tau protein in both physiological and pathological conditions. Physiol Rev.

[B46] Gould TD, Manji HK (2005). Glycogen synthase kinase-3: a putative molecular target for lithium mimetic drugs. Neuropsychopharmacology.

[B47] Ryves WJ, Harwood AJ (2001). Lithium inhibits glycogen synthase kinase-3 by competition for magnesium. Biochem Biophys Res Commun.

[B48] Grimes CA, Jope RS (2001). The multifaceted roles of glycogen synthase kinase 3β in cellular signaling. Prog Neurobiol.

[B49] Harwood AJ (2005). Lithium and bipolar mood disorder: the inositol-depletion hypothesis revisited. Mol Psychiatry.

[B50] Wada A, Yokoo H, Yanagita T, Kobayashi H (2005). Lithium: potential therapeutics against acute brain injuries and chronic neurodegenerative diseases. J Pharmacol Sci.

[B51] Nunes PV, Forlenza OV, Gattaz WF (2007). Lithium and risk for Alzheimer's disease in elderly patients with bipolar disorder. Br J Psychiatry.

[B52] Quintanilla RA, Munoz FJ, Metcalfe MJ, Hitschfeld M, Olivares G, Godoy JA, Inestrosa NC (2005). Trolox and 17β-estradiol protect against amyloid β-peptide neurotoxicity by a mechanism that involves modulation of the *Wnt* signaling pathway. J Biol Chem.

[B53] Alvarez AR, Godoy JA, Mullendorff K, Olivares GH, Bronfman M, Inestrosa NC (2004). *Wnt-3a* overcomes β-amyloid toxicity in rat hippocampal neurons. Exp Cell Res.

[B54] Busciglio J, Lorenzo A, Yeh J, Yankner BA (1995). β-amyloid fibrils induce tau phosphorylation and loss of microtubule binding. Neuron.

[B55] Takashima A, Honda T, Yasutake K, Michel G, Murayama O, Murayama M, Ishiguro K, Yamaguchi H (1998). Activation of tau protein kinase I/glycogen synthase kinase-3β by amyloid β peptide (25–35) enhances phosphorylation of tau in hippocampal neurons. Neuroscience research.

[B56] Sheldahl LC, Slusarski DC, Pandur P, Miller JR, Kuhl M, Moon RT (2003). Dishevelled activates Ca2+ flux, PKC, and CamKII in vertebrate embryos. J Cell Biol.

[B57] De Ferrari GV, Moon RT (2006). The ups and downs of *Wnt* signaling in prevalent neurological disorders. Oncogene.

[B58] Esposito G, Scuderi C, Lu J, Savani C, De Filippis D, Iuvone T, Steardo L, Sheen V, Steardo L (2008). S100B induces tau protein hyperphosphorylation via Dickopff-1 up-regulation and disrupts the *Wnt* pathway in human neural stem cells. J Cell Mol Med.

[B59] Mudher A, Lovestone S (2002). Alzheimer's disease-do tauists and baptists finally shake hands?. Trends Neurosci.

[B60] Jones SE, Jomary C, Grist J, Stewart HJ, Neal MJ (2000). Modulated expression of secreted frizzled-related proteins in human retinal degeneration. Neuroreport.

[B61] Jones SE, Jomary C, Grist J, Stewart HJ, Neal MJ (2000). Altered expression of secreted frizzled-related protein-2 in retinitis pigmentosa retinas. Invest Ophthalmol Vis Sci.

[B62] Rattner A, Hsieh JC, Smallwood PM, Gilbert DJ, Copeland NG, Jenkins NA, Nathans J (1997). A family of secreted proteins contains homology to the cysteine-rich ligand-binding domain of frizzled receptors. Proc Natl Acad Sci USA.

[B63] Melkonyan HS, Chang WC, Shapiro JP, Mahadevappa M, Fitzpatrick PA, Kiefer MC, Tomei LD, Umansky SR (1997). SARPs: a family of secreted apoptosis-related proteins. Proc Natl Acad Sci USA.

[B64] Baranski M, Berdougo E, Sandler JS, Darnell DK, Burrus LW (2000). The dynamic expression pattern of frzb-1 suggests multiple roles in chick development. Dev Biol.

[B65] Strittmatter WJ, Roses AD (1995). Apolipoprotein E and Alzheimer disease. Proc Natl Acad Sci USA.

[B66] Caruso A, Motolese M, Iacovelli L, Caraci F, Copani A, Nicoletti F, Terstappen GC, Gaviraghi G, Caricasole A (2006). Inhibition of the canonical *Wnt* signaling pathway by apolipoprotein E4 in PC12 cells. J Neurochem.

[B67] Daw EW, Payami H, Nemens EJ, Nochlin D, Bird TD, Schellenberg GD, Wijsman EM (2000). The number of trait loci in late-onset Alzheimer disease. Am J Hum Genet.

[B68] de Vrij FM, Fischer DF, van Leeuwen FW, Hol EM (2004). Protein quality control in Alzheimer's disease by the ubiquitin proteasome system. Prog Neurobiol.

[B69] Oddo S (2008). The ubiquitin-proteasome system in Alzheimer's disease. J Cell Mol Med.

[B70] Liu C, Li Y, Semenov M, Han C, Baeg GH, Tan Y, Zhang Z, Lin X, He X (2002). Control of β-catenin phosphorylation/degradation by a dual-kinase mechanism. Cell.

[B71] Aberle H, Bauer A, Stappert J, Kispert A, Kemler R (1997). β-catenin is a target for the ubiquitin-proteasome pathway. EMBO J.

[B72] Fuenzalida K, Quintanilla R, Ramos P, Piderit D, Fuentealba RA, Martinez G, Inestrosa NC, Bronfman M (2007). Peroxisome proliferator-activated receptor gamma up-regulates the Bcl-2 anti-apoptotic protein in neurons and induces mitochondrial stabilization and protection against oxidative stress and apoptosis. J Biol Chem.

[B73] Farias GG, Valles AS, Colombres M, Godoy JA, Toledo EM, Lukas RJ, Barrantes FJ, Inestrosa NC (2007). *Wnt-7a* induces presynaptic colocalization of α7-nicotinic acetylcholine receptors and adenomatous polyposis coli in hippocampal neurons. J Neurosci.

[B74] Colombres M, Cerpa W, Velarde V, Nibaldo CI (2008). Insulin degrading enzyme (IDE) is a target gene of the *Wnt* signaling pathway (In Prep). FEBS Lett.

[B75] Arrazola M, Varela-Nallar L, Colombres M, Assar R, Aravena A, Maass A, Martínez S, Inestrosa NC (2008). Calcium/calmodulin-dependent protein kinase type IV (CaMKIV) is a target gene of the *Wnt*/β-catenin signaling pathway. EMBO Rep.

[B76] Inestrosa N, Colombres M, Assar R, Hodar C, Aravena A, Gonzalez M, Maass A, Martinez S (2008). Detection of Novel *Wnt* Target in the Human Genome. Mol Psychiatry.

[B77] Li H-L, Wang H-H, Liu S-J, Deng Y-Q, Zhang Y-J, Tian Q, Wang X-C, Chen X-Q, Yang Y, Zhang J-Y (2007). Phosphorylation of tau antagonizes apoptosis by stabilizing β-catenin, a mechanism involved in Alzheimer's neurodegeneration. PNAS.

[B78] Wang Y, Thekdi N, Smallwood PM, Macke JP, Nathans J (2002). Frizzled-3 is required for the development of major fiber tracts in the rostral CNS. J Neurosci.

[B79] Lyuksyutova AI, Lu C-C, Milanesio N, King LA, Guo N, Wang Y, Nathans J, Tessier-Lavigne M, Zou Y (2003). Anterior-Posterior Guidance of Commissural Axons by *Wnt*-Frizzled Signaling. Science.

[B80] Wang Y, Huso D, Cahill H, Ryugo D, Nathans J (2001). Progressive cerebellar, auditory, and esophageal dysfunction caused by targeted disruption of the frizzled-4 gene. J Neurosci.

[B81] Zhao C, Aviles C, Abel RA, Almli CR, McQuillen P, Pleasure SJ (2005). Hippocampal and visuospatial learning defects in mice with a deletion of frizzled 9, a gene in the Williams syndrome deletion interval. Development.

[B82] Wang Y, Guo N, Nathans J (2006). The role of Frizzled3 and Frizzled6 in neural tube closure and in the planar polarity of inner-ear sensory hair cells. J Neurosci.

[B83] Sancho E, Batlle E, Clevers H (2004). Signaling pathways in intestinal development and cancer. Annu Rev Cell Dev Biol.

[B84] Polakis P (2007). The many ways of *Wnt* in cancer. Curr Opin Genet Dev.

[B85] Wang HY, Malbon CC (2004). *Wnt*-frizzled signaling to G-protein-coupled effectors. Cell Mol Life Sci.

[B86] Karasawa T, Yokokura H, Kitajewski J, Lombroso PJ (2002). Frizzled-9 is activated by *Wnt-2* and functions in *Wnt*/β-catenin signaling. J Biol Chem.

[B87] Caricasole A, Ferraro T, Iacovelli L, Barletta E, Caruso A, Melchiorri D, Terstappen GC, Nicoletti F (2003). Functional characterization of *WNT7A* signaling in PC12 cells: interaction with A FZD5 × LRP6 receptor complex and modulation by Dickkopf proteins. J Biol Chem.

[B88] Gazit A, Yaniv A, Bafico A, Pramila T, Igarashi M, Kitajewski J, Aaronson SA (1999). Human frizzled 1 interacts with transforming *Wnts* to transduce a TCF dependent transcriptional response. Oncogene.

[B89] Carmon KS, Loose DS (2008). *Wnt7a* interaction with Fzd5 and detection of signaling activation using a split eGFP. Biochem Biophys Res Commun.

[B90] Shimogori T, VanSant J, Paik E, Grove EA (2004). Members of the *Wnt*, Fz, and Frp gene families expressed in postnatal mouse cerebral cortex. J Comp Neurol.

[B91] Lein ES, Hawrylycz MJ, Ao N, Ayres M, Bensinger A, Bernard A, Boe AF, Boguski MS, Brockway KS, Byrnes EJ (2007). Genome-wide atlas of gene expression in the adult mouse brain. Nature.

[B92] Chacon MA, Varela-Nallar L, Inestrosa NC (2008). Frizzled-1 is involved in the neuroprotective effect of *Wnt3a* against Aβ oligomers. Journal of Cellular Physiology.

[B93] Ferreira ST, Vieira MN, De Felice FG (2007). Soluble protein oligomers as emerging toxins in Alzheimer's and other amyloid diseases. IUBMB Life.

[B94] Magdesian MH, Nery AA, Martins AH, Juliano MA, Juliano L, Ulrich H, Ferreira ST (2005). Peptide blockers of the inhibition of neuronal nicotinic acetylcholine receptors by amyloid β. J Biol Chem.

[B95] Geula C, Mesulam MM (1994). Cholinergic systems and related neuropathological predilection patterns in Alzheimer's disease.

[B96] Haring R, Gurwitz D, Barg J, Pinkas-Kramarski R, Heldman E, Pittel Z, Wengier A, Meshulam H, Marciano D, Karton Y (1994). Amyloid precursor protein secretion via muscarinic receptors: reduced desensitization using the M1-selective agonist AF102B. Biochem Biophys Res Commun.

[B97] Genis I, Fisher A, Michaelson DM (1999). Site-specific dephosphorylation of tau of apolipoprotein E-deficient and control mice by M1 muscarinic agonist treatment. J Neurochem.

[B98] Xie J, Guo Q, Zhu H, Wooten MW, Mattson MP (2000). Protein kinase C iota protects neural cells against apoptosis induced by amyloid β-peptide. Brain Res Mol Brain Res.

[B99] Caccamo A, Oddo S, Billings LM, Green KN, Martinez-Coria H, Fisher A, LaFerla FM (2006). M1 Receptors Play a Central Role in Modulating AD-like Pathology in Transgenic Mice. Neuron.

[B100] Vlad SC, Miller DR, Kowall NW, Felson DT (2008). Protective effects of NSAIDs on the development of Alzheimer disease. Neurology.

[B101] Citron M (2004). Strategies for disease modification in Alzheimer's disease. Nat Rev Neurosci.

[B102] Morihara T, Chu T, Ubeda O, Beech W, Cole GM (2002). Selective inhibition of Aβ 42 production by NSAID R-enantiomers. J Neurochem.

[B103] Weggen S, Eriksen JL, Das P, Sagi SA, Wang R, Pietrzik CU, Findlay KA, Smith TE, Murphy MP, Bulter T (2001). A subset of NSAIDs lower amyloidogenic Aβ 42 independently of cyclooxygenase activity. Nature.

[B104] Zhou Y, Su Y, Li B, Liu F, Ryder JW, Wu X, Gonzalez-DeWhitt PA, Gelfanova V, Hale JE, May PC (2003). Nonsteroidal anti-inflammatory drugs can lower amyloidogenic Aβ 42 by inhibiting Rho. Science.

[B105] Farias GG, Godoy JA, Vazquez MC, Adani R, Meshulam H, Avila J, Amitai G, Inestrosa NC (2005). The anti-inflammatory and cholinesterase inhibitor bifunctional compound IBU-PO protects from β-amyloid neurotoxicity by acting on *Wnt* signaling components. Neurobiol Dis.

[B106] Miranda S, Opazo C, Larrondo LF, Munoz FJ, Ruiz F, Leighton F, Inestrosa NC (2000). The role of oxidative stress in the toxicity induced by amyloid β-peptide in Alzheimer's disease. Prog Neurobiol.

[B107] Cerpa W, Godoy JA, Alafaro I, Farias GG, Metcalfe MJ, Fuentealba R, Bonansco C, Inestrosa NC (2008). *WNT-7a* modulates the synaptic vesicle cycle and synaptic transmission in hippocampal neurons. J Biol Chem.

[B108] Behl C, Davis JB, Lesley R, Schubert D (1994). Hydrogen peroxide mediates amyloid β protein toxicity. Cell.

[B109] Santos MJ, Quintanilla RA, Toro A, Grandy R, Dinamarca MC, Godoy JA, Inestrosa NC (2005). Peroxisomal proliferation protects from β-amyloid neurodegeneration. J Biol Chem.

[B110] Willson TM, Wahli W (1997). Peroxisome proliferator-activated receptor agonists. Curr Opin Chem Biol.

[B111] Watson GS, Craft S (2003). The role of insulin resistance in the pathogenesis of Alzheimer's disease: implications for treatment. CNS Drugs.

[B112] Watson GS, Craft S (2006). Insulin resistance, inflammation, and cognition in Alzheimer's Disease: lessons for multiple sclerosis. J Neurol Sci.

[B113] Landreth GE, Heneka MT (2001). Anti-inflammatory actions of peroxisome proliferator-activated receptor γ agonists in Alzheimer's disease. Neurobiol Aging.

[B114] Feinstein DL (2003). Therapeutic potential of peroxisome proliferator-activated receptor agonists for neurological disease. Diabetes Technol Ther.

[B115] Inestrosa NC, Godoy JA, Quintanilla RA, Koenig CS, Bronfman M (2005). Peroxisome proliferator-activated receptor γ is expressed in hippocampal neurons and its activation prevents β-amyloid neurodegeneration: role of *Wnt* signaling. Exp Cell Res.

[B116] Fuentealba RA, Farias G, Scheu J, Bronfman M, Marzolo MP, Inestrosa NC (2004). Signal transduction during amyloid-β-peptide neurotoxicity: role in Alzheimer disease. Brain Res Brain Res Rev.

[B117] Camacho IE, Serneels L, Spittaels K, Merchiers P, Dominguez D, De Strooper B (2004). Peroxisome Proliferator-Activated Receptor {γ} Induces a Clearance Mechanism for the Amyloid-{β} Peptide. J Neurosci.

[B118] Pedersen WA, Flynn ER (2004). Insulin resistance contributes to aberrant stress responses in the Tg2576 mouse model of Alzheimer's disease. Neurobiol Dis.

[B119] Role LW, Berg DK (1996). Nicotinic receptors in the development and modulation of CNS synapses. Neuron.

[B120] Levin ED, Simon BB (1998). Nicotinic acetylcholine involvement in cognitive function in animals. Psychopharmacology (Berl).

[B121] Vernino S, Amador M, Luetje CW, Patrick J, Dani JA (1992). Calcium modulation and high calcium permeability of neuronal nicotinic acetylcholine receptors. Neuron.

[B122] Banerjee C, Nyengaard JR, Wevers A, de Vos RA, Jansen Steur EN, Lindstrom J, Pilz K, Nowacki S, Bloch W, Schroder H (2000). Cellular expression of α7 nicotinic acetylcholine receptor protein in the temporal cortex in Alzheimer's and Parkinson's disease – a stereological approach. Neurobiol Dis.

[B123] Kem WR (2000). The brain α7 nicotinic receptor may be an important therapeutic target for the treatment of Alzheimer's disease: studies with DMXBA (GTS-21). Behav Brain Res.

